# Elucidation of binding mechanism of hydroxyurea on serum albumins by different spectroscopic studies

**DOI:** 10.1186/2193-1801-3-360

**Published:** 2014-07-15

**Authors:** Keerti M Naik, Deepa B Kolli, Sharanappa T Nandibewoor

**Affiliations:** P. G. Department of Studies in Chemistry, Karnatak University, Dharwad, 580 003 India

**Keywords:** Hydroxyurea, Bovine serum albumin, Human serum albumin, Fluorescence

## Abstract

**Objectives:**

The interaction of hydroxyurea (HU) with serum albumins (SAs) has not been investigated so far. However, it necessitates the interaction study of HU with SAs in phosphate buffer of pH 7.4.

**Methods:**

The binding of HU on bovine serum albumin (BSA) and human serum albumin (HSA) was studied in vitro under simulated physiological conditions by spectroscopic methods viz., fluorescence, FT-IR, UV–vis absorption, synchronous fluorescence and three-dimensional fluorescence.

**Results:**

The Stern-Volmer plot indicated the presence of dynamic quenching mechanism in the interaction of HU with SAs. The number of binding sites, n and binding constants, K were obtained at various temperatures according to the double logarithm regression curve. The result of FT-IR spectra, UV–vis absorption, synchronous fluorescence and three-dimensional fluorescence spectra showed that the conformation of SAs has been changed in the presence of HU. The thermodynamic parameters were calculated according to van’t Hoff equation and discussed.

**Conclusion:**

This kind of study of interaction between BSA and HSA with HU would be useful in pharmaceutical industry, life sciences and clinical medicine.

## Introduction

**Figure 1:**
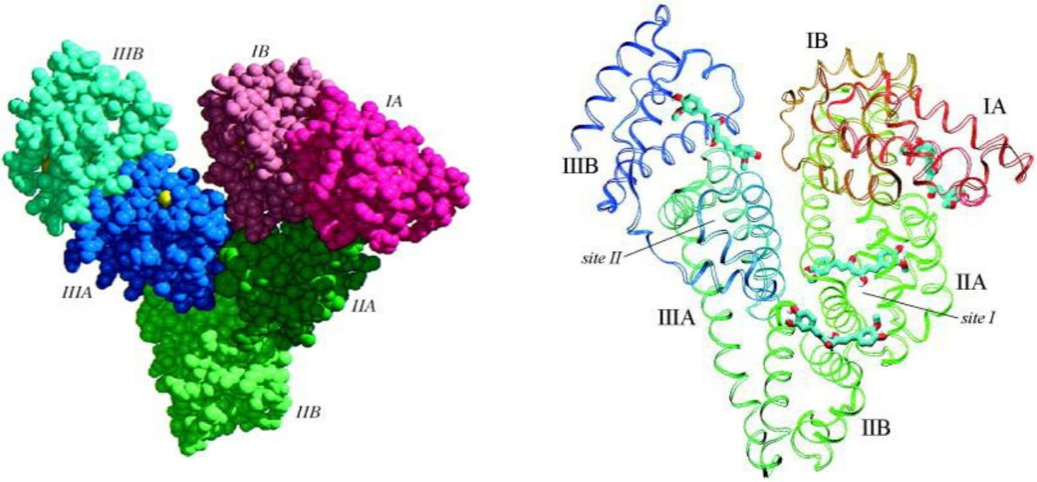
**Structure of BSA and HSA.**

It is known that the distribution, free concentration and the metabolism of various drugs are strongly affected by drug–protein interactions in the blood stream (Hu et al. 2005; Kamat 2005; Naik et al. 2009). This type of interaction can also influence the drug stability and toxicity during the chemotherapeutic process (Hu et al. 2005). Serum albumin is the most abundant protein present in the circulatory system of a wide variety of organisms and being major macromolecule it contributes to osmotic blood pressure (Carter & Ho 1994). It can play a dominant role in drug disposition and efficiency (Olson & Christ 1996). Many drugs and other bioactive small molecules bind reversibly to albumin and other serum components, which then function as carriers. Serum albumin often increases the apparent solubility of hydrophobic drugs in plasma and modulates their delivery to cell in vivo and in vitro. Animal experiments are indispensable in providing basic information on the pharmacological actions, biotransformation and biodistribution of drugs (He & Carter 1992; Guo et al. 2004). Bovine serum albumin (BSA) (Figure 1) is well suited to these initial studies, since it has been extensively characterized (Guo et al. 2004). Similarly human serum albumin (HSA) (Figure 1) as the most abundant carrier protein plays an important role in the transport and disposition of many endogenous and exogenous substances such as metabolites, drugs, and other biologically active compounds present in blood (Carter & Ho 1994). Recently, its three-dimensional (3D) structure has been determined through X-ray crystallographic measurements (Carter & Ho 1994) and consists of three structurally homologous domains which assemble to form a heart-shaped molecule, each domain contains two sub-domains. Serum albumin binds and transports many ligands, including fatty acids, amino acids, hormones, cations, anions, and variety of pharmaceuticals. It is suggested that the principal regions of ligand binding to HSA are located in hydrophobic cavities in the sub-domains IIA and IIIA, which are consistent with sites I and II, respectively, and single tryptophan residue of HSA is in subdomain IIA (Carter & Ho 1994; He & Carter 1992). In addition, drug–albumin complex may be considered as a model for gaining general fundamental insights into drug–protein binding. Plasma protein binding of drugs assumes great importance since it influences their pharmacokinetic and pharmacodynamic properties, and may also cause interference with the binding of other endogenous and/or exogenous ligands as a result of overlap of binding sites and/or conformational changes. Therefore, detailed investigation of drug–protein interaction assumes significance for thorough understanding of the pharmacokinetic behavior of a drug and for the design of analogues with effective pharmacological properties. Fluorescence quenching is a useful method to study the reactivity of chemical and biological systems since it allows non-intrusive measurements of substances in low concentration under physiological conditions (Naik et al. 2010; Gu et al. 2007). It can reveal accessibility of quenchers to serum albumins fluorophores, help to understand serum albumins binding mechanisms to compounds and provides clues to the nature of the binding phenomenon. Fourier transform infrared (FT-IR spectroscopy), a powerful technique for the study of hydrogen bonding, has recently become very popular for structural characterization of proteins. The most important advantage of FT-IR spectroscopy for biological studies is that spectra of almost any biological system can be obtained in a wide verity of environments.

**Scheme 1:**
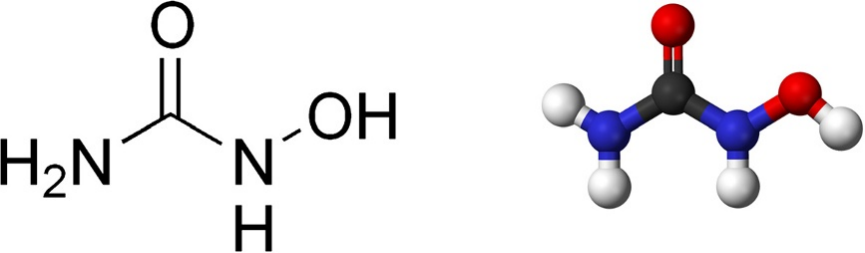
Structural formula of hydroxyurea. **Structural formula of hydroxyurea.**

Hydroxyurea (HU), the simplest, 1-carbon organic antitumor agent, is a member of the substituted urea group and is chemically known as hydroxycarbamide (Donehower 1990). In 1981 it was reported to have antineoplastic activity against sarcoma (Van Belle et al. 1986). At present, the primary role of hydroxyurea (Scheme 1) in chemotherapy is the management of granulocytic leukemia and thrombocytosis. It has been used in combination with radiotherapy for carcinomas of the head and neck (Heerenberg 1992). HU is used in the treatment of cancer (Donehower 1992), sickle cell anemia (Charache et al. 1992) and infection with the human immunodeficiency virus (HIV) (Gao et al. 1993). HU is a potent, nonalkylating myelosuppressive agent that inhibits DNA synthesis (Krakoff et al. 1968).

The interaction of HU with SAs has not been investigated so far. However, it necessitates the interaction study of HU with SAs in phosphate buffer of pH 7.4. Different aspects of HU-serum albumin interactions viz., quenching mechanism, binding force operating between the drug and proteins, the distance of separation between the protein and HU (based on the theory of fluorescence resonance energy transfer), conformational changes etc. have been studied. This is the first report on the mechanism of interaction of HU with SAs employing fluorescence spectroscopy, UV–vis absorption, FT-IR, synchronous fluorescence and three-dimensional fluorescence spectroscopic methods.

### Experimental section

#### Reagents and chemicals

Bovine serum albumin (BSA) and human serum albumin (HSA) were purchased from Sigma Chemical Company, St. Louis, USA and used without purification. Hydroxyurea (HU) was obtained from Sigma Aldrich. The solutions of HU, BSA and HSA were prepared in 0.1 M phosphate buffer of pH 7.4 with respect to their molecular weight. All other materials were of analytical reagent grade and Millipore water was used throughout the work.

### Instrumentation used

Fluorescence spectra were recorded using a RF-5301 PC Hitachi spectrofluorometer Model F-2000 (Tokyo, Japan) with a 150 W Xenon lamp, a 1 cm quartz cell and thermostatic cuvette holder. The excitation and emission bandwidths were both 5 nm. The temperature of the sample was maintained by recycling water throughout the experiment. The absorption spectra were recorded on a singlet beam CARY 50-BIO UV–vis. Spectrophotometer (Victoria, Australia), FT-IR Nicolet-5700 (USA) was used to record infrared spectra. All of the pH measurements were performed with an Elico LI120 pH meter (Elico Ltd., India).

### Procedures

#### Hydroxyurea with protein interaction study

A stock solution of 250 μM of BSA, HSA and HU were prepared in phosphate buffer solution (pH 7.4). An appropriate volume of BSA or HSA to obtain 5 μM and 5 μM HU was mixed and fluorescence spectra were recorded. In the next step fixing the concentration of HSA at 5 μM and drug concentration was varied from 5 to 45 μM. Fluorescence spectra were recorded at three different temperatures (288, 298 and 308 K) in the range 300–450 nm upon excitation at 295 and 280 nm in BSA and HSA case.

### Absorption measurements

The UV measurements of BSA and HSA in the presence and absence of HU were made in the range of 240–340 nm. BSA and HSA concentration was fixed at 5 μM while the drug concentration was varied from 5 to 10 μM in presence of phosphate buffer at 298 K.

### FT-IR measurements

The FT-IR spectra of BSA and HSA in presence and absence of HU at 298 K were recorded in the range of 1600 – 3000 cm^-1^. Serum albumins concentration was fixed at 5 μM while that of HU was 20 μM in presence of phosphate buffer.

### Synchronous fluorescence measurements

The synchronous fluorescence characteristics of HU-SAs were noted down at different scanning intervals of Δλ (Δλ = λ_em_ - λ_ex_). When Δλ = 15 nm, the spectrum characteristics of protein tyrosine residues were observed and when Δλ = 60 nm, the spectrum characteristics of protein tryptophan residues were noticed.

### 3-D fluorescence studies

3-D fluorescence spectrum was recorded under the following conditions: excitation wavelength range of 250–350 nm and emission wavelength range of 200–500 nm and an increment of 10 nm with other parameters were just the same as that of fluorescence quenching spectra. C_protein_ = 5 μM and C_HU_ = 20 μM.

### Effects of some common ions

The effects of some common ions viz., Co^2+^, Cu^2+^, Ni^2+^, Ca^2+^ and Zn^2+^ were investigated on HU with BSA and HSA interactions. The fluorescence spectra of HU with BSA or HSA system were recorded in presence of above ions at excitation at 295 and 280 nm for BSA and HSA at 298 K. The overall concentration of BSA or HSA and that of the common ions were fixed at 5 μM, while the concentration of HU was varied from 0 to 45 μM at 298 K.

## Results and discussion

### Analysis of fluorescence quenching of serum albumins by hydroxyurea

**Figure 2:**
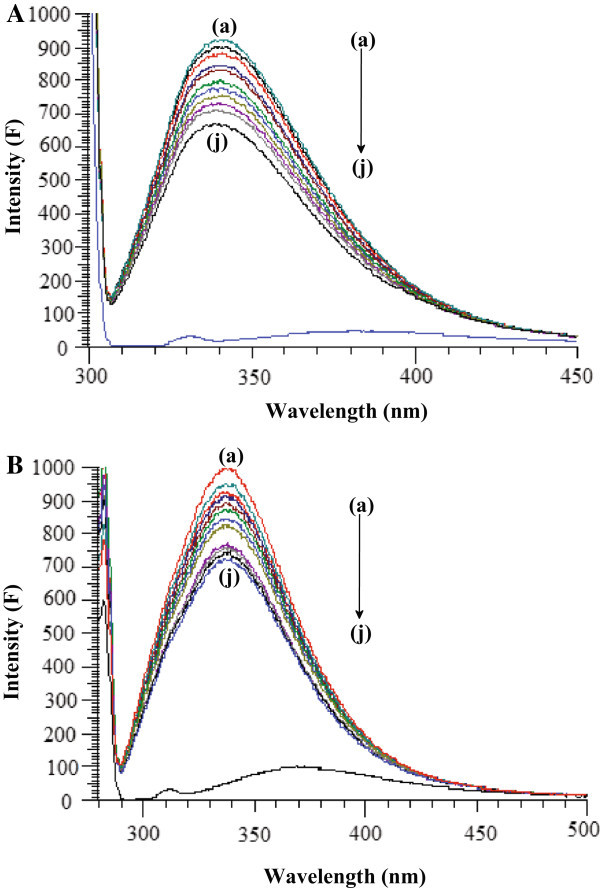
**Fluorescence spectra of BSA and HSA. A.** Fluorescence spectra of BSA (5 μM) in presence of HU: (a) 0 μM, (b) 5 μM, (c) 10 μM, (d) 15 μM, (e) 20 μM, (f) 25 μM, (g) 30 μM, (h) 35 μM, (i) 40 μM, (j) 45 μM. **B.** Fluorescence spectra of HSA (5 μM) in presence of HU: (a) 0 μM, (b) 5 μM, (c) 10 μM, (d) 15 μM, (e) 20 μM, (f) 25 μM, (g) 30 μM, (h) 35 μM, (i) 40 μM, (j) 45 μM.

Fluorescence quenching is the decrease of the quantum yield of fluorescence from a fluorophore induced by a variety of molecular interactions with a quencher molecule. Generally, the fluorescence of serum albumins comes from tryptophan, tyrosine and phenylalanine residues. The intrinsic fluorescence of BSA and HSA is almost due to tryptophan alone because phenylalanine has a very low quantum yield and the fluorescence of tyrosine is almost totally quenched if it is ionized or near an amino group, a carboxyl group or a tryptophan. This viewpoint was well supported by the experimental observations of Sulkowska (Sulkowska 2002). That is, the change of intrinsic fluorescence intensity of BSA and HSA was due to tryptophan residue when small molecules bound to BSA and HSA. When different amounts of drug solution were titrated with a fixed concentration of SAs, a remarkable decrease in the fluorescence intensity of SAs was observed with hydroxyurea (Figure 2) in the present study. Under the experimental conditions, hydroxyurea did not show any fluorescence intensity. A blue shift in maximum emission wavelength of BSA and HSA observed upon the addition of hydroxyurea was probably due to the loss of the compact structure of hydrophobic sub-domain where tryptophan was placed (Sulkowska 2002).

### Binding parameters and mechanism

Quenching can be induced by dynamic and static process. Dynamic and static quenching can be distinguished based on their differences on temperature dependence. Higher temperature results in faster diffusion and larger amounts of collisional quenching. It will typically lead to the dissociation of weakly bound complexes and smaller amounts of static quenching. Therefore, the quenching constant increases for dynamic quenching while it decreases for static quenching with increase in temperature. In order to invoke this possible quenching mechanism, the fluorescence quenching data were subjected to Stern–Volmer analysis using the equation: (Lakowicz 1999)
1

where F and F_o_ are the fluorescence intensity of BSA or HSA with and without quencher (drug), respectively. K_q_, K_SV_, Γ_o_ and [Q] are the quenching rate constant of the biomolecule, the dynamic quenching constant, the average lifetime of the biomolecule without quencher and the concentration of quencher, respectively. Obviously,
2

**Figure 3:**
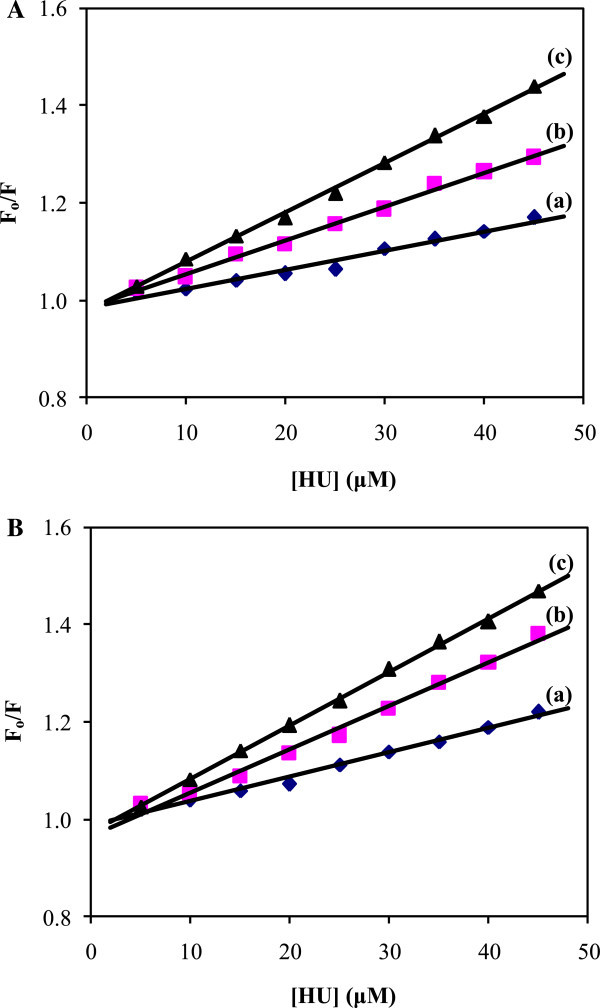
**The Stern–Volmer plots with** **BSA and HSA. A.** The Stern–Volmer plots for quenching of HU with BSA at 288 K (a), 298 K (b) and 308 K (c). **B.** The Stern–Volmer plots for quenching of HU with HSA at 288 K (a), 298 K (b) and 308 K (c).

The Stern–Volmer plots for representative HU–SAs systems are shown in Figure 3. The Stern–Volmer plots were observed to be linear in both BSA and HSA with HU. The slopes were increasing in both HU-BSA and HU-HSA systems with increase in temperature. The values of K_SV_ at different temperatures were evaluated and are given in Tables 1 and 2. The values of K_SV_ at different temperatures indicated that the presence of dynamic quenching mechanism in both the interaction between BSA and HSA with HU.

**Table 1:** **Interaction parameters of HU**-**BSA system at different temperatures**

Temp. (K)	Ksv × 10 ^-4^ M ^-1^	Kq × 10 ^-12^ M ^-1^S ^-1^	Binding constant K × 10 ^-4^ M ^-1^	No. of binding sites n	ΔH ^0^ (k J mol ^-1^)	ΔS ^0^ (J K ^-1^ mol ^-1^)	ΔG ^0^ (k J mol ^-1^)
288	0.3966	0.3966	1.5049	1.15			
298	0.6941	0.6941	3.1754	1.15	58.08 ± 2.0	281.47 ± 13.0	-25.79 ± 3.0
308	1.0150	1.0150	7.2795	1.19			

**Table 2:** **Interaction parameters of HU**-**HSA system at different temperatures**

Temp. (K)	Ksv × 10 ^-4^ M ^-1^	Kq × 10 ^-12^ M ^-1^S ^-1^	Binding constant K × 10 ^-4^ M ^-1^	No. of binding sites n	ΔH ^0^ (k J mol ^-1^)	ΔS ^0^ (J K ^-1^ mol ^-1^)	ΔG ^0^ (k J mol ^-1^)
288	0.5042	0.5042	1.1601	1.09			
298	0.8949	0.8949	4.3863	1.17	102.94 ± 3.0	434.95 ± 12.0	-26.67 ± 2.0
308	1.1019	1.1019	18.958	1.28			

Since the fluorescence lifetime of the biopolymer (Chen et al. 1990) is 10^-8^ s, the quenching rate constant, K_q_ can be calculated using the above equation. The values of K_q_ are given in Tables 1 and 2. The maximum scatter collision quenching constant, K_q_ of various quenchers with the biopolymer (Lakowicz & Weber 1973) is reported to be 2 × 10 (Donehower 1990) LM^-1^ S^-1^. The order of magnitude of K_q_ was calculated to be 10 (Heerenberg 1992) for both BSA and HSA with HU systems in the present study. So, the rate constants of the protein quenching procedure initiated by HU are greater than the value of K_q_ for the scatter mechanism.

### Binding parameters

Fluorescence intensity data can also be used to obtain the binding constant, K and the number of binding sites, n. When small molecules bind independently to a set of equivalent sites on a macromolecule, the equilibrium between free and bound molecules is given (Wang et al. 2007) by the following equation.
3

**Figure 4:**
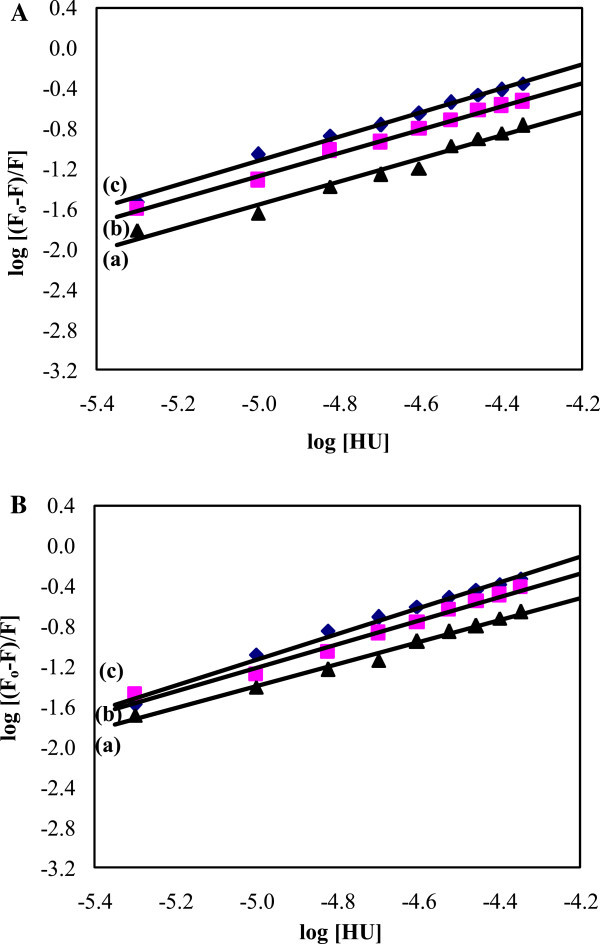
**The plot of log of BSA and HSA. A.** The plot of log (Fo-F)/F vs log [Q] for quenching of BSA by HU at 288 K (a), 298 K (b) and 308 K (c). **B.** The plot of log (Fo-F)/F vs log [Q] for quenching of HSA by HU at 288 K (a), 298 K (b) and 308 K (c).

The values of K and n were obtained from the intercept and slope of the plot of log [(F_o_ - F)/F] vs. log [Q] (Figure 4) and are given in Tables 1 and 2. The value of K is significant to understand the distribution of the drug in plasma since the weak binding can lead to a short lifetime or poor distribution, while strong binding can decrease the concentrations of free drug in plasma. It was noticed that the binding constant values increased with the increase in temperature in HU and BSA or HSA interactions. These results indicate that there is a higher binding affinity and much more stable complex between HU with BSA or HSA. The value of n is helpful to know the number of binding sites and to locate the binding site in BSA and HSA for the drug. The values of n for HU with BSA and HSA were noticed to be almost unity indicating that there were one independent class of binding sites on BSA and HSA with HU. Hence, the HU most likely bound to the hydrophobic pocket located in sub-domain IIA; that is to say Trp 214 is near or within the binding site (Hong et al. 2004).

### Thermodynamic parameters and the nature of binding forces

The thermodynamic parameters, free energy change (ΔG^0^), enthalpy change (ΔH^0^) and entropy change (ΔS^0^) of HU with BSA and HSA interaction are important for confirming binding mode. For this purpose, the temperature dependence of binding constant was studied. Binding studies were carried out at 288, 298 and 308K at which SAs does not undergo any structural degradation. The molecular forces contributing to protein interactions with small molecular substrates may include van-der Waals interactions, hydrogen bonds, electrostatic and hydrophobic interactions and so on (Ulrich 1981). The thermodynamic parameters were evaluated using the van’t Hoff equation and Gibbs–Helmholtz equation:
45

**Figure 5:**
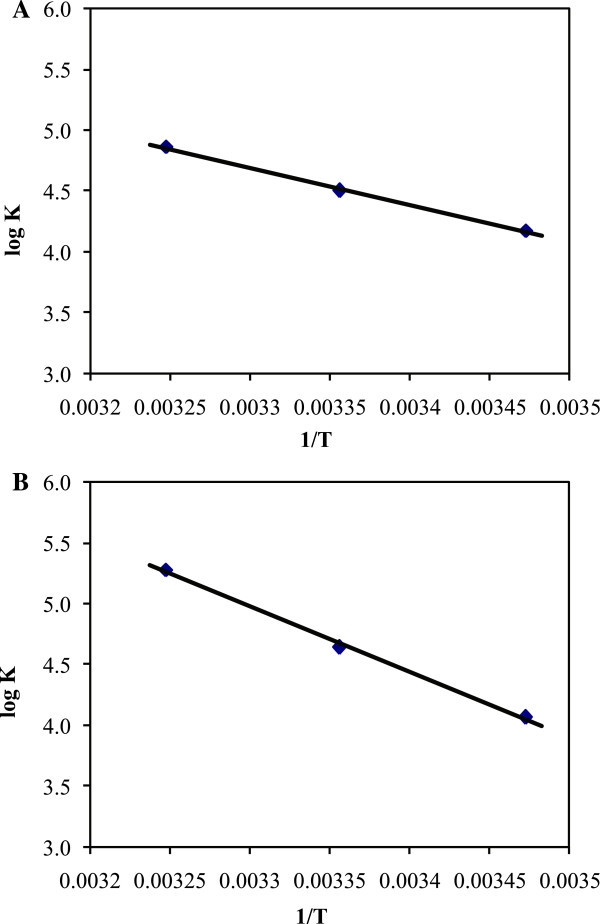
**Van’t Hoff plot with BSA and HSA. A.** van’t Hoff plot for the binding of HU with BSA. **B.** van’t Hoff plot for the binding of HU with HSA.

The plot of log K versus 1/T (Figure 5) enabled the determination of the values of ΔH^0^ and ΔS^0^. Ross and Subramanian (Ross & Subramanian 1981) have characterized the sign and magnitude of the thermodynamic parameters associated with various individual kinds of interaction. For typical hydrophobic interactions, both ΔH^0^ and ΔS^0^ are positive, while these are negative for van-der Waals forces and hydrogen-bond formation in low dielectric media (Ross & Subramanian 1981; Mallick et al. 2005). Moreover, the specific electrostatic interaction between ionic species in an aqueous solution is characterized by positive ΔS^0^ value and negative ΔH^0^ value (small). For HU–SA complex, the main source of ΔG^0^ value was derived from a large contribution of ΔS^0^ term with a little contribution from ΔH^0^ factor. So, the main interaction between HU–SAs was believed to be hydrophobic. However, the electrostatic interaction could not be excluded. Negative values of ΔG^0^ obtained in both the cases indicated the spontaneity of interaction.

### Absorption spectroscopic studies

**Figure 6:**
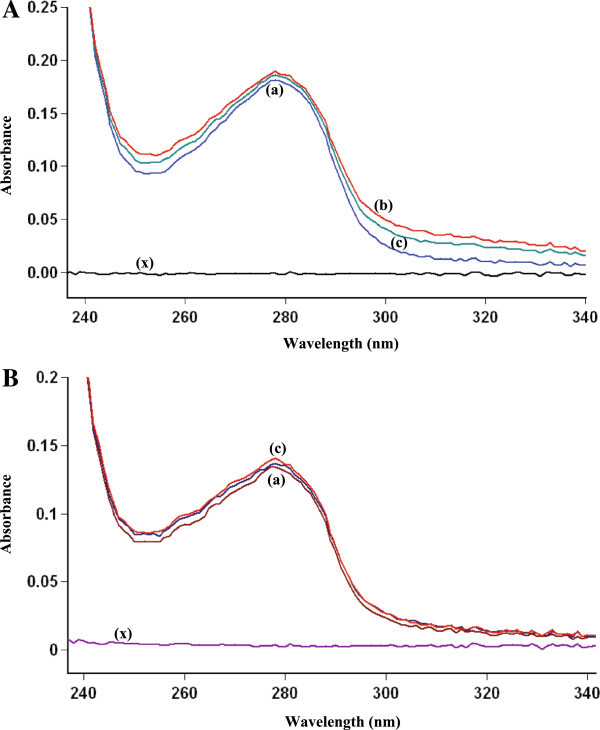
**Absorbance spectra of BSA and HSA. A.** Absorbance spectra of BSA, HU and HU-BSA system. BSA concentration was 5 μM (a). HU concentration for HU–BSA system was at 5 μM (b) and 10 μM (c). (x) is the concentration of 5 μM HU. **B.** Absorbance spectra of HSA, HU and HU-HSA system. HSA concentration was 5 μM (a). HU concentration for HU–HSA system was at 5 μM (b) and 10 μM (c). (x) is the concentration of 5 μM HU.

UV–vis absorption measurement is a very simple method and applicable to know the change in hydrophobicity (Wang et al. 2007) and to know the complex formation (Valeur 2001). In the present study, we have observed the change in UV absorption spectra of BSA, HSA and HU–SAs systems (Figure 6). Due to the shift in λ_max_, a formation of complex between HU and serum albumins (Bi et al. 2005) was suggested. It is evident that the UV absorption intensity of serum albumins increased regularly with the increase in concentration of HU. The change in λ_max_ also indicates the change in polarity around the tryptophan residue and the change in peptide strand of BSA and HSA molecule and hence the change in hydrophobicity (Shaikh et al. 2007).

### FT-IR spectroscopic studies

**Figure 7:**
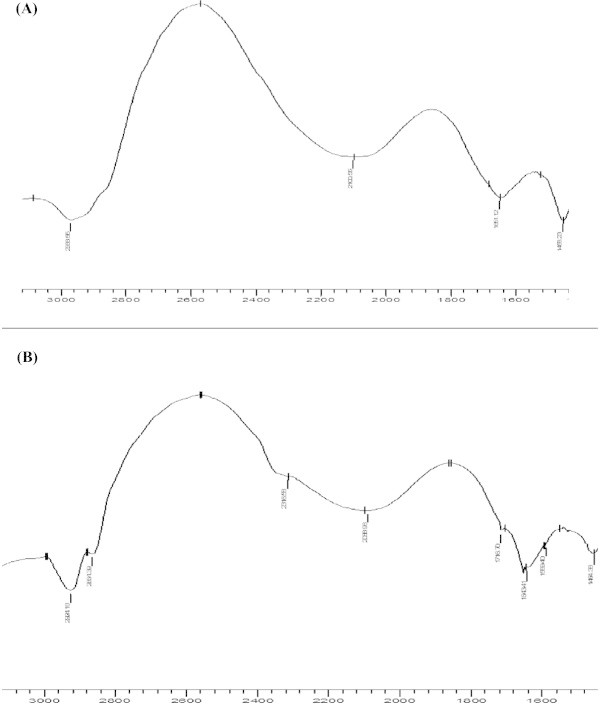
**FT-** **IR spectra of (A) BSA-** **HU system and (B) HSA-** **HU system.**

Additional evidence for HU-SAs interactions were obtained from FT-IR spectra. Infrared spectrum of protein exhibited a number of amide bands due to different vibrations of the peptide moiety. Of all the amide modes of the peptide group, the single most widely used one in studies of protein secondary structure is the amide I. The amides I and II peaks occurred in the region of 1600 – 1700 cm^-1^ and 1500 – 1600 cm^-1^, respectively (Figure 7). Amide I band is more sensitive to changes in protein secondary structure compared to amide II. Hence, the amide I is more useful for studies of secondary structure (Wi et al. 1998; Rahmelow & Hubner 1996). The FT-IR spectrum reveals that the peak position of amide I was shifted from 1641 to1651 cm^-1^ in the IR spectrum of BSA upon interaction with HU and 1651 to1643 cm^-1^ in the IR spectrum of HSA upon interaction with HU. This indicated that the HU interacted with BSA and HSA and the secondary structure of SAs was changed.

### Synchronous fluorescence spectra

**Figure 8:**
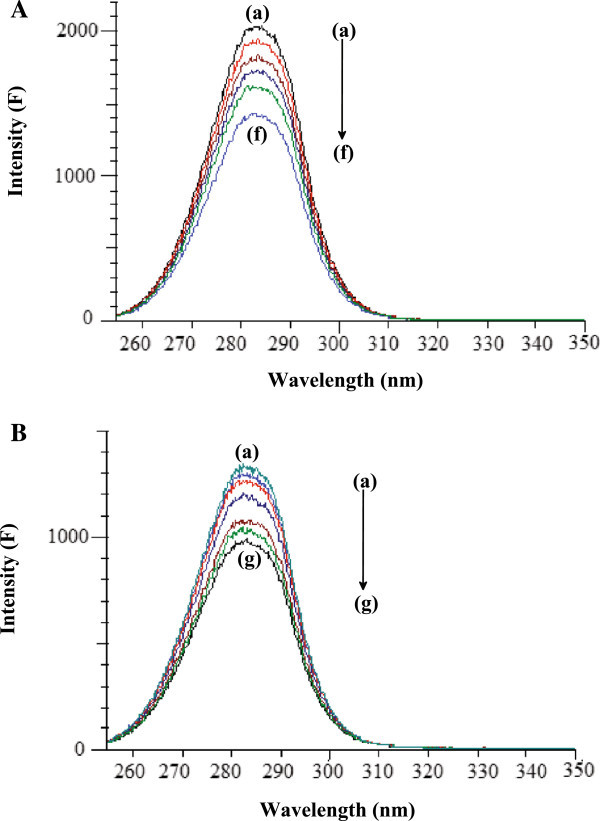
**Synchronous fluorescence spectra of BSA and HSA. A.** Synchronous fluorescence spectra of BSA-HU: For Δλ = 60 nm. Concentration of HU: (a) 0, (b) 5, (c) 10, (d) 15, (e) 20 and (f) 25 μM. The concentration of BSA was 5.0 μM. **B.** Synchronous fluorescence spectra of HSA-HU: **(A)** For Δλ = 60 nm. Concentration of HU: (a) 0, (b) 5, (c) 10, (d) 15, (e) 20, (f) 25 and (g) 30 μM. The concentration of HSA was 5.0 μM.

Synchronous fluorescence spectra provide information on the molecular environment of the fluorophore functional group. The value of Δλ i.e. difference between excitation and emission wavelengths is an important operating parameter. According to Miller (Miller 1979) when Δλ is 15 nm, synchronous fluorescence spectra indicates the changes in the microenvironment of tyrosine residues and when Δλ is 60 nm, it provides information on the microenvironment of tryptophan residues. With the unchanged concentration of the BSA, HSA and the concentration of HU increased by titration, the synchronous spectroscopy were performed at Δλ = 15 nm and Δλ = 60 nm and are shown in Figure 8 (only Δλ = 60 nm were given).

It can be seen from the Figure 8, the emission strength of tryptophan residues decreased faster than that of tyrosine residues, which revealed that tryptophan residues contributed more to the quenching of intrinsic BSA and HSA florescence in both the system. In addition, a slight red shift observed in both the tyrosine and tryptophan residues, indicated the less hydrophobic environment and more exposed to the solvent molecules during the binding process in both the systems. So both the microenvironment of tyrosine and tryptophan residues was changed, resulting in conformational changes of BSA and HSA during the binding process (Liu et al. 2001; Zhang et al. 2008).

### Three-dimensional fluorescence spectra

**Figure 9:**
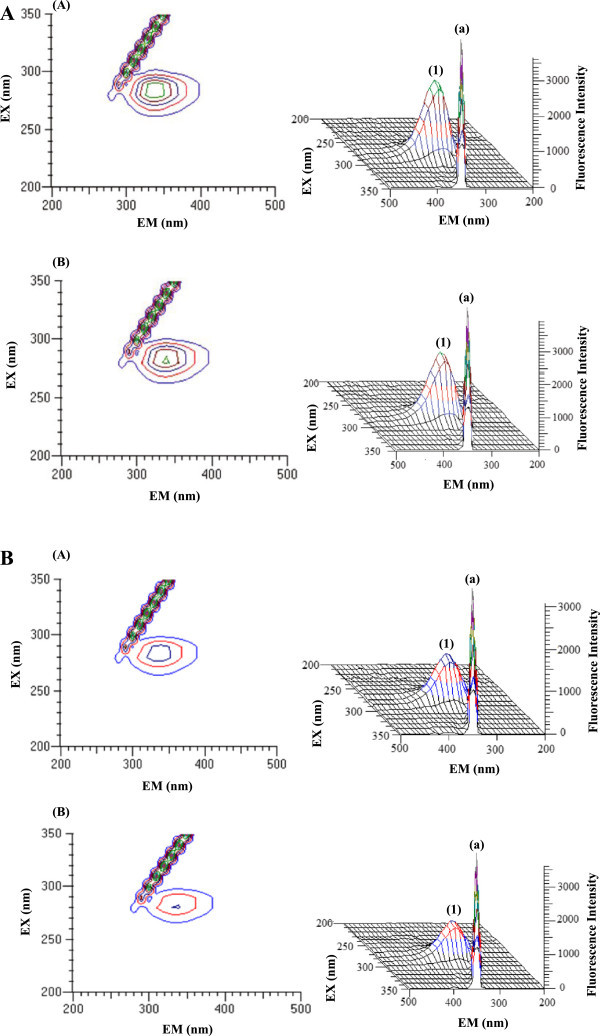
**The 3**-**D fluorescence spectra of BSA and HSA. A.** The 3-D fluorescence spectra and corresponding contour diagrams of BSA **(A)** and BSA-HU **(B).** The concentration of protein was 5 μM and that of HU was 20 μM. **B.** The 3-D fluorescence spectra and corresponding contour diagrams of HSA **(A)** and HSA-HU **(B)**. The concentration of protein was 5 μM and that of HU was 20 μM.

Three-dimensional fluorescence spectra have become a popular fluorescence analysis technique in recent years (Weber 1961). It is well known that three-dimensional fluorescence spectrum can provide more detailed information about the change of the configuration of proteins. In addition, the contour map can also provide a lot of important information. Figure 9A presents the three-dimensional fluorescence spectra and contour ones of BSA (A) and BSA-HU (B) and Figure 9B presents the three-dimensional fluorescence spectra and contour ones of HSA (A) and HSA-HU (B), respectively. The contour map displayed a bird’s eye view of the fluorescence spectra. In both the Figures 9A and B, peak a is the Rayleigh scattering peak (λ_ex_ = λ_em_) (Zhang & Mei 2009). With the addition of HU, the fluorescence intensities of peak a increased. The reason for this is that when the HU-SAs complex was formed, it caused the diameter of the macromolecule to increase which in turn resulted in enhanced scattering effects.

As referred to peak 1, it mainly reveals the spectral characteristic of tryptophan and tyrosine residues. The reason is that when serum albumin is excited at 280 nm for BSA and 280 nm for HSA, it mainly reveals the intrinsic fluorescence of tryptophan and tyrosine residues, while fluorescence by the phenylalanine (Phe) residue is negligible. Compared with UV absorption spectrum of SAs (Figure 6), there is an absorption peak at around 295 nm for BSA and 280 nm for HSA, which is mainly induced by the π → π * transition of an aromatic amino acid. The Trp, Tyr and Phe residues in the binding cavity of protein have conjugated π - electrons and thus easily form charge transfer compounds with electron deficient species or other π - electron systems (Kang et al. 2004). The fluorescence intensity of the peak 1 decreased markedly and the maximum emission wavelengths of the peak 1 have obvious blue shift following the addition of HU, indicating that the conformations of the tryptophan and tyrosine residues of BSA and HSA were altered. Therefore, we can conclude that the binding of HU-SAs induced some micro environmental and conformational changes in BSA and HSA, a complex between HU-SAs has been formed.

### Effect of metal ions on the interactions of serum albumins by hydroxyurea

In plasma, there are some metal ions, which can affect the interactions of the drugs and serum albumins. Trace metal ions, especially the bivalent type are essential in the human body and play an important structural role in many proteins. It is reported (Kang et al. 2004) that Cu^2+^, Zn^2+^, Ni^2+^, Co^2+^ and Ca^2+^ and other metal ions can form complexes with serum albumins. Hence, the effects of some metal salt solutions viz., CuCl_2_, ZnCl_2_, NiCl_2_, CoCl_2_ and CaCl_2_ on the binding of HU with BSA and HSA were investigated in the present study. Under the experimental conditions, none of the cations gave the precipitate in phosphate buffer. The binding constants of HU with BSA and HSA in presence of above ions were evaluated and the results are shown in Table 3. The binding constant of HU-BSA system decreased in presence of Cu^2+^, Ca^2+^, Zn^2+^ and Ni^2+^, whereas in HU-HSA system, the binding constant decreased in presence of Co^2+^, Ca^2+^, Zn^2+^ and Ni^2+^. This was likely to be caused by a conformational change in the vicinity of the binding site. The decrease in the binding constant in presence of above metal ions would shorten the storage time of the drug in blood plasma and hence more amount of free drug would be available in plasma (Shaikh et al. 2007). This led to the need for more doses of drug to achieve the desired therapeutic effect in presence of above ions. The binding constant increased in presence of Co^2+^ in HU-BSA system, while Cu^2+^ in the HU-BSA system which indicates the formation of metal ion-drug complex via metal ion bridge. This led to the need for less dose of drug for desired therapeutic effect. This may prolong storage period of HU in blood plasma and enhance its maximum effects.

**Table 3:** **Effect of common ions on binding constant of HU**-**BSA and HU**-**HSA systems**

Systems	Binding constant (M ^-1^)	
	BSA	HSA
HU	3.1754 × 10^4^	4.3863 × 10^4^
HU + Co^2+^	2.08 × 10^5^	4.18 × 10^4^
HU + Ni^2+^	2.74 × 10^4^	3.74 × 10^4^
HU + Ca^2+^	3.13 × 10^4^	3.83 × 10^4^
HU + Zn^2+^	2.98 × 10^4^	4.08 × 10^4^
HU + Cu^2+^	2.86 × 10^4^	3.26 × 10^5^

### Comparison of two systems

The values of binding constants (Table 1) suggest the interaction of HU with BSA and HSA are almost similar fashion. The quenching mechanism is also similar in both the system i.e., dynamic quenching. The binding sites were unity in both the systems. The thermodynamic parameters revealed that the HU and BSA and HSA undergo hydrophobic interaction. The synchronous fluorescence spectra and three dimensional fluorescence spectra reveals that the microenvironment of tyrosine and tryptophan residues was changed, resulting in conformational changes of SAs during the binding process.

## Conclusions

The present work provides an approach for studying the interactions of BSA and HSA with hydroxyurea using absorption, fluorescence, FT-IR, synchronous and 3-D fluorescence techniques under physiological conditions. The results showed that BSA and HSA fluorescence was quenched by HU through dynamic quenching mechanism. HU interacted with SAs through hydrophobic forces. The remarkable change of amide I peak position in the BSA and HSA infrared spectrum after interaction with HU indicated that secondary structure of BSA and HSA has been changed. Since, the pharmaceutical firms need standardized screens for protein binding in the first step of new drug design, this kind of study of interaction between BSA and HSA with HU would be useful in pharmaceutical industry, life sciences and clinical medicine.
